# Scalable Fabrication of Highly Flexible Porous Polymer-Based Capacitive Humidity Sensor Using Convergence Fiber Drawing

**DOI:** 10.3390/polym11121985

**Published:** 2019-12-02

**Authors:** Maryam Mesgarpour Tousi, Yujing Zhang, Shaowei Wan, Li Yu, Chong Hou, Ning Yan, Yoel Fink, Anbo Wang, Xiaoting Jia

**Affiliations:** 1 Bradley Department of Electrical and Computer Engineering, Virginia Polytechnic Institute and State University, Blacksburg, VA 24060, USA; maryam2c@vt.edu (M.M.T.); yujingz1@vt.edu (Y.Z.); shaowei@vt.edu (S.W.); yuli@vt.edu (L.Y.); ning112@vt.edu (N.Y.); awang@vt.edu (A.W.); 2 School of Optical and Electronic Information, Huazhong University of Science & Technology, Wuhan 430074, China; chong@hust.edu.cn; 3 Research Laboratory of Electronics, Massachusetts Institute of Technology, Cambridge, MA 02139, USA; yoel@mit.edu; 4 Institute for Soldier Nanotechnologies, Massachusetts Institute of Technology, Cambridge, MA 02139, USA; 5 Department of Materials Science and Engineering, Massachusetts Institute of Technology, Cambridge, MA 02139, USA

**Keywords:** capacitive humidity sensor, convergence fiber drawing, porous polymer, flexible, PEI

## Abstract

In this study, we fabricated a highly flexible fiber-based capacitive humidity sensor using a scalable convergence fiber drawing approach. The sensor’s sensing layer is made of porous polyetherimide (PEI) with its porosity produced in situ during fiber drawing, whereas its electrodes are made of copper wires. The porosity induces capillary condensation starting at a low relative humidity (RH) level (here, 70%), resulting in a significant increase in the response of the sensor at RH levels ranging from 70% to 80%. The proposed humidity sensor shows a good sensitivity of 0.39 pF/% RH in the range of 70%–80% RH, a maximum hysteresis of 9.08% RH at 70% RH, a small temperature dependence, and a good stability over a 48 h period. This work demonstrates the first fiber-based humidity sensor fabricated using convergence fiber drawing.

## 1. Introduction

Humidity control is a common concern for many industrial applications such as automobile manufacturing [1], meteorology [2], agriculture [3], food processing [4], aerospace [5], medicine [6], and biotechnology [7]. Significant progress was made in monitoring, detecting, and controlling the ambient humidity under varying conditions [8]. Nevertheless, how to manufacture cost-effective, scalable, flexible yet robust and strong fiber-based humidity sensors that can be used in wearable electronics, smart textiles, and personal and environmental monitoring remains a challenging task.

Based on their operating principles, the existing humidity sensors can be broadly categorized into three groups: capacitive [9,10,11,12], resistive [13,14,15,16,17,18], and optical sensors [19,20,21,22,23]. Among them, the capacitive sensors are the most commonly used in the industrial and commercial fields [2] due to their high sensitivity and performance, linearity, fast response time [24,25,26], low power consumption, and good stability at higher humidity levels [12]. These sensors detect the humidity variations by changing the sensing layer dielectric when exposed to the different atmospheric humidity conditions. Their total capacitance values vary with the amount of water vapor uptake.

The capacitive humidity sensors are either ceramic-based [27,28,29] or polymer-based [30,31,32,33,34,35,36]. Each type has its own advantages. The ceramic-based capacitive humidity sensors have mechanical strength, thermal capabilities, and quick response time [2], yet lack flexibility. The polymer-based ones, on the other hand, have good hygroscopic property, flexibility [37], and long-term stability [2]. Furthermore, they are easy to fabricate at low cost [22]. A wide range of conjugated polymer materials such as poly(phenylene) (PP) [38], poly(p-phenylenevinylene) (PPV) [39], and polythiophene (PTh) [40] can be utilized in capacitive polymer-based humidity sensors.

In this paper, we propose a new type of polymer-based capacitive humidity sensor produced using a scalable fiber drawing method. The sensor is highly flexible and cost-effective. More importantly, its structural and mechanical properties make it ideal in many unique applications such as wearable electronics or functional fabrics [41,42].

In a polymer-based capacitive humidity sensor, water vapor from the sensor’s surrounding environment can be absorbed by the hygroscopic polymer and occupy the space between the polymer molecules. As a result, the dielectric permittivity of the polymer changes linearly with the change in the amount of water absorbed [5]. In order to absorb water easily and be reusable, the organic polymers used as the sensing layer of the capacitive humidity sensors have to be at least partly hygroscopic, as well as hydrophobic [31,32]. Polyetherimide (PEI), a special class of polyimide (PI) [43,44], for instance, possesses such properties and is, therefore, used in this study. PEI contains cyclic imide in its back bone, and demonstrates exceptional mechanical, thermal, and electrical properties [45].

Despite their great characteristics, organic polymers, including PEI, have some weaknesses such as low response to humidity variation and large hysteresis. The latter may result in the malfunction of the sensor due to the polymer deformation. In order to overcome these drawbacks, reports showed that the sensitivity and sensing response of polymer-based capacitance humidity sensors can be significantly improved by manipulating the porosity of a sensor’s polymer sensing layer [46,47]. This is because the polymeric sensor’s response is directly proportional to the diffusion of analyte into the sensing layer [48]. Typically, hydrophilic polymers are not suitable candidates for being employed in the structure of humidity sensors due to their solubility in water. In this regard, using porous and crosslinked polymer films typically fabricated via a graft polymerization method rather than nonporous-structure polymers is a known and efficient way to overcome the issue of hydrophilicity of some polymer materials such as poly(2-acryl-amido-2-methylpropanesulfonate) or poly(2-hydroxy-3-methacryloxypropyi trimethylammonium chloride) [49,50]. For instance, the sensor developed by Jiang et al. [51], based on LiCl (Lithium chloride)-loaded microporous polymer, demonstrated high sensitivity, minute hysteresis, improved durability, and fast response/recovery. Drawing on this development, in this paper, we propose a novel capacitive relative humidity (RH) sensor with porous PEI as the sensing layer, which was fabricated using a convergence fiber drawing approach.

The multimaterial fiber thermal drawing process is a very promising process that can enable complex and multiple functionalities within fibers of various shapes and textures [52]. Recent development in the fabrication of advanced functional fibers led to optical, electrical, and chemical sensing devices with unprecedented properties [53,54,55,56,57,58]. However, existing fiber fabrication methods are either based on high-temperature material drawing (silica, silicon, gold, and copper) or low-temperature material drawing (polymer with low-melting-point metals). To achieve the flexibility, multifunctionality, and electrical performance of both types of materials, it is critical to converge these two processes into one scalable fabrication process.

Here, we report a non-conventional fiber drawing method, the convergence fiber drawing method, which allows for high-temperature metal electrodes (copper, *T*_m_ = 1085 °C) and low-temperature polymer (PEI, *T*_g_ = 210 °C) to be drawn simultaneously into a single device. We chose copper in this study because of its superior electrical performance. We used PEI not only for its special aforementioned properties, but also because its glass transition temperature is compatible with our draw tower working temperature range. Furthermore, we introduced porosity in the PEI during the fiber drawing, which significantly increased the sensitivity of the humidity sensor in the higher-RH regime due to the capillary condensation effect.

Our proposed fiber-based humidity sensor is cost-effective, easy to fabricate, and flexible, and it has long-term stability. It can withstand significant bending over a prolonged period of time. Its sensitivity is largely independent of the temperature variation and is compatible with the integrated circuit (IC) fabrication technology. With its unique properties, our sensor can be useful in many applications ranging from soil moisture measurement in agriculture to interior moisture control in architecture, wearable real-time moisture monitoring for medical and health purposes, and the remote controlling of the atmosphere RH in places that require the device being flexible.

## 2. Materials and Methods

### 2.1. Porous Fiber Fabrication Using the Thermal Drawing Process

The materials which were used in this paper to fabricate porous fiber and the porous polymer-based capacitive humidity sensor included PEI rods (McMaster-Carr, outer diameter (OD) = 6.35 mm, 19.05 mm, 25.4 mm), polyphenylsulfone(PPSU) tubes (McMaster-Carr, OD = 25.4 mm, inner diameter (ID) = 19.05 mm; OD = 31.75 mm, ID = 19.05 mm), and copper wires (McMaster-Carr, OD = 0.127 mm). The fabrication process was carried out using a thermal drawing process (TDP) with a custom-made fiber draw tower. The imaging of the fabricated fiber cross-section was done by SEM (LEO 1550 field-emission SEM, Zeiss, Oberkochen, Germany) and optical microscope (Le Croy-DDA-260, Teledyne LeCroy, Chestnut Ridge, NY, USA). The mechanical characterization of the sensor was evaluated using a differential mechanical analyzer (DMA) (Q800, TA Instruments, New Castle, DE, USA). To investigate the performance of the sensor, a custom-made testing chamber consisting of an environmental chamber (Test Equity 1000-series environmental test chamber, Test Equity, Moorpark, CA, USA), a commercial digital air humidity controller sensor (IMAGE WH8040, Willhi, Shenzhen, Guangdong, China), a humidifier (Honeywell HUL520W, Honeywell, Charlotte, NC, USA), and two dehumidifiers (Ivation IVAGDM36, Ivation, Edison, NJ, USA) was used. The measurements were carried out via a CMOS (Complementary metal–oxide–semiconductor) 555 connected to an Oscilloscope (Axiovert 25, Zeiss, Oberkochen, Germany).

Macroscopic polymer-based preforms can be drawn into hundreds of meters of thin fibers with the scaled down geometry of the original preform using a thermal drawing process (TDP) [54]. Using TDP, we fabricated porous fibers from polymer preforms with absorbed water.

In the TDP method, the preform is exposed to three different temperatures inside the furnace. The middle zone temperature of the furnace, which plays the most important role in the fiber fabrication process, is highest among all the three zones, and must be above the *T*_g_ of the polymer. For the case of PEI (McMaster-Carr, Douglasville, GA, USA), which has a *T*_g_ of 210 °C, the middle zone temperature is typically set between 320 and 360 °C to ensure a stable drawing process. Polymer preforms become viscous when they reach the middle zone of the furnace, and are ready to be pulled into fibers. When we draw polymer preforms that contain absorbed water, the water evaporates and leaves behind pores in the polymer when the preform’s temperature is above the *T*_g_ of the polymer. By applying a continuous stress when pulling down the preform, which is now soft and porous, we obtain a fiber full of interconnected pores. We previously reported the fabrication of porous poly(methylmethacrylate) and poly(carbonate) polymer fibers using this method [59]. In order to determine the parameters that affect the fabrication of porous polymer fiber, we also investigated the relationship between the weight percentage (wt %) of water inside the polymer and the porosity in the fabricated fiber, and the effect of the bottom zone temperature on the size and distribution of pores in the fiber.

**Figure 1:**
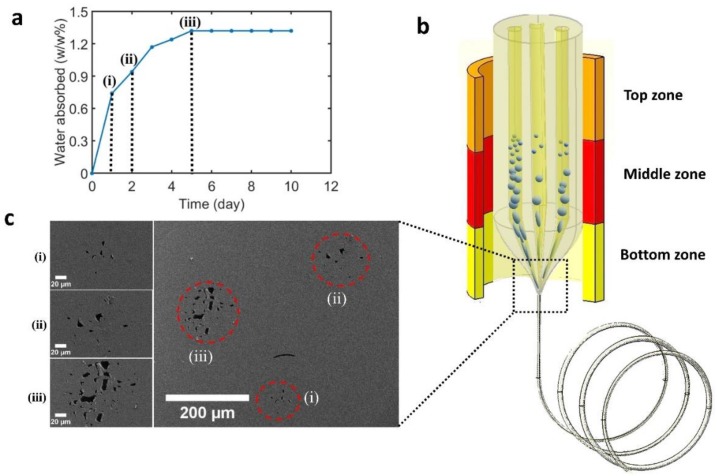
Porous polymer fiber fabrication using the thermal drawing process (TDP). (**a**) The increase in the absorbed water weight percentile after soaking polyetherimide (PEI) rods in the 60 °C water bath for 1–10 days. (i), (ii), and (iii) correspond to one, two, and five days of soaking, respectively. (**b**) Schematic fabrication process of a PEI preform containing three PEI rods with different water weight percentages (as indicated by (i), (ii), and (iii) in (**a**)). (**c**) SEM images of the fiber cross-section with three different regions (red circles in the image) of porosity corresponding to the different water weight percentages in the preform prior to drawing.

To understand how the weight percentage of water in the preform affects the porosity generated in the fiber, we firstly prepared three 6.35 mm outer-diameter (OD) baked PEI rods. We stored them in an oven under vacuum with a temperature of 165 °C for a week to remove the residual water in the as-received rods. We then measured the weight of these baked PEI rods, which we called the “dry weight”. After baking, we soaked the rods in a 60 °C water bath for 10 days and recorded the increase in the weight of these rods every day, as shown in Figure 1a. The PEI rods became saturated after five days of soaking in the water bath. The final weight of the PEI rods increased by 0.74%, 0.94%, and 1.32%, compared to their dry weight, after one day, two days, and five days of soaking, respectively, as indicated by (i), (ii), and (iii) in Figure 1a.

To prepare the preform, we machined three holes with a diameter of 6.35 mm inside the 25.4 mm OD PEI rod, which was already dried under vacuum at 168 °C for three weeks. Then, we placed the three 6.35 mm OD rods with varying water weight percentage into these three holes. Afterward, the preform was drawn into a fiber with the OD between 500 and 900 µm using the TDP. The top, middle, and bottom temperatures (see Figure 1b) during the TDP were 205, 342, and 180 °C, respectively.

Figure 1c shows the cross-sectional SEM images of the fiber that incorporated three different water weight percentage regions (0.74%, 0.94%, and 1.32%). The SEM images show that the PEI rods with higher initial water weight percentage prior to thermal drawing exhibited larger and more interconnected pores after drawing, although the distribution of pores within all three regions appeared random. It is worth mentioning that, due to the thick cladding in this structure, the pore sizes were smaller in comparison with our humidity sensor device which had a much thinner cladding, allowing the water vapor to expand more at evaluated temperatures.

We then investigated the effect of the cooling rate on the size and distribution of the pores generated in the fiber. The cooling rate of the TDP is controlled by the bottom zone temperature of the furnace. The lower the bottom zone temperature is, the higher the cooling rate of the fiber is. We used a PEI rod (OD = 19.05 mm) with water weight percentage of 1.12%, which was placed into a baked PPSU tube (McMaster-Carr, ID = 19.05 mm, OD = 25.4 mm) to ensure a stable drawing. Due to the fact that the glass transition temperature of PPSU (*T*_g_ = 220 °C) [60] is close enough to that of PEI (*T*_g_ = 210 °C), they can be drawn simultaneously using TDP. The preform was drawn into a fiber using three different bottom zone temperatures of 195, 180, and 150 °C with the same middle zone temperature of 342 °C.

Figure S1 (Supplementary Materials) shows the optical images of the resultant fiber cross-sections. It demonstrates that the bottom zone temperature played a key role in the produced pore size and distribution. The pores of the fiber drawn under the lowest bottom temperature (150 °C) were much smaller and more uniform than those drawn at 180 and 195 °C.

### 2.2. Sensor Fabrication Using the Convergence Fiber Drawing Process

**Figure 2:**
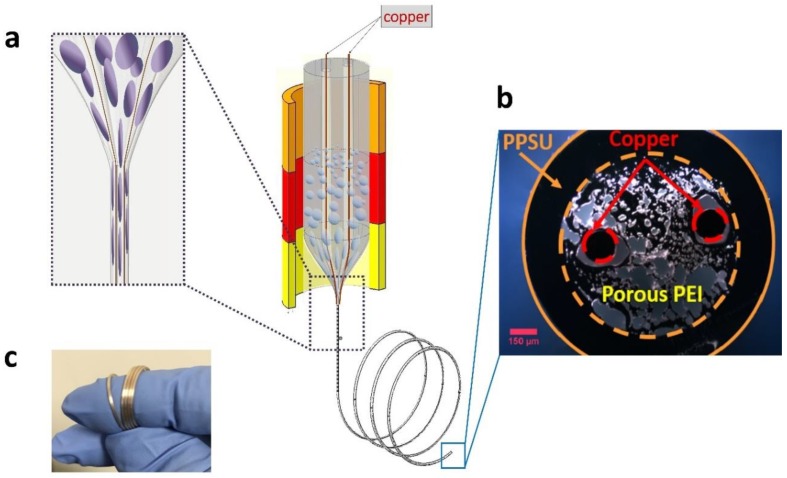
Fiber capacitive humidity sensor fabrication using the convergence fiber drawing method. (**a**) An illustration of convergence fiber drawing process. (**b**) Optical microscope image of the cross-section of the fabricated humidity sensor. (**c**) Photograph of the drawn fiber.

We designed and fabricated a fiber-based capacitive humidity sensor using porous PEI as the sensing layer, and two embedded copper wires as the electrodes. In order to draw the polymer (PEI), which has a relatively low *T*_g_, simultaneously with the metal (copper), which has a relatively high melting point (*T*_m_), we developed a convergence fiber drawing process. The convergence fiber drawing process involves continuous feeding of the metal wire through a hollow channel in the polymer preform during the TDP. The polymer preform is scaled down during the TDP, together with the hollow channel, until the diameter of the hollow channel matches that of the metal wire, resulting in the convergence drawing of the polymer and metal materials (Figure 2a).

Our macroscopic preform consisted of a PEI rod with 1.12 wt % absorbed water, surrounded by a baked PPSU tube which served as a sacrificial cladding. Two hollow channels (3 mm in diameter, with a spacing of 9.5 mm) were machined in the PEI rode to host two copper wires (0.127 mm in diameter). The PEI rod had an OD of 19.05 mm. The PPSU tube had an inner diameter (ID) of 19.05 mm, and an OD of 31.75 mm. This vacuum-dried PPSU cladding was used to protect the fiber’s final geometry from deformation caused by the pore generation process during the TDP. During the TDP, we set the middle and bottom zone temperatures at 340 and 165 °C, respectively. The middle and bottom zone temperatures were chosen to enable a stable and uniform draw of the porous PEI and copper wires. At higher temperatures, the pores tend to collapse, causing the deformation of the final structure in the fiber. At lower temperatures, the tension increases, and the porous PEI tends to break during the TDP. The fiber was drawn at a draw-down ratio of 23.6, resulting in a fiber diameter of ~1 mm. Figure 2b shows an optical image of the cross-section of the fabricated fiber. Finally, we removed the PPSU sacrificial cladding using an optical-fiber stripper, and the OD of the fiber sensor decreased to 0.7 mm. The fabricated fiber sensor was highly flexible, as demonstrated in Figure 2c.

## 3. Results

### 3.1. Mechanical and Electrical Capacitive Performance

**Figure 3:**
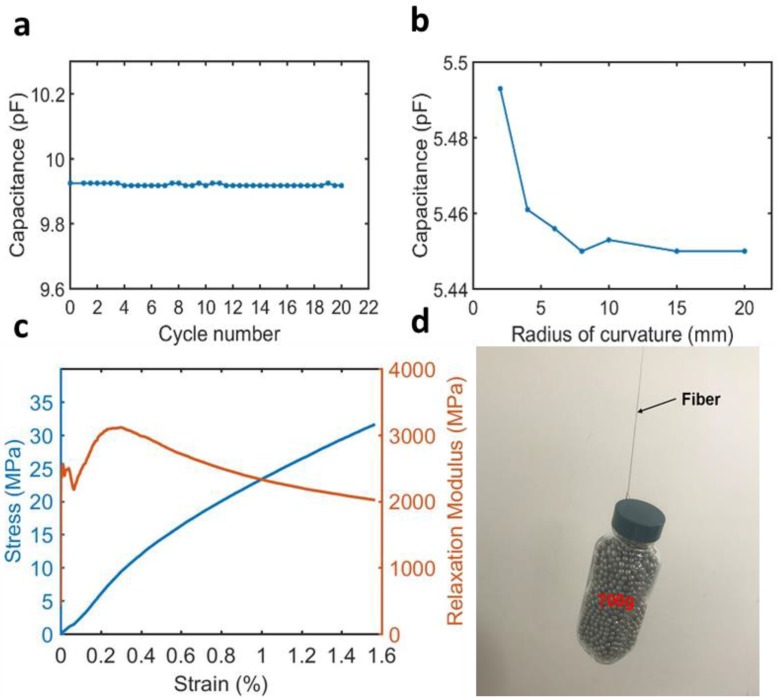
Mechanical tests for the flexible fiber capacitive humidity sensor. (**a**) The effect of bending cycles on the performance of the sensor with the bending radius fixed at 6.5 mm. An extremely small capacitance change was observed during 20 bending cycles. (**b**) The effect of bending radius on the performance of the sensor. (**c**) Stress–strain curve (blue) and relaxation modulus curve (orange) of flexible fiber capacitive humidity sensor. (**d**) A weight (700 g) was lifted using a single-fiber humidity sensor.

In order to test the performance of the fiber sensor upon repeated bending, we measured the capacitance change of the fiber sensor (length = 20 cm) using a custom-made bending test stage at room temperature (24 °C), and an RH level of 16%. The capacitance value was consistent after 20 cycles of bending at a 6.5 mm radius of curvature and a 180° bending angle (Figure 3a). We then tested the stability of the sensor under varying bending radii at a bending angle of 90°. The sensor’s capacitance value was consistent until the bending radius was reduced to 4 mm. Afterward, deviation of the capacitance value was observed at a bending radius below 4 mm (Figure 3b). These results indicate that the fiber sensor can withstand significant bending over a prolonged period of time.

To evaluate the mechanical strength of the fiber humidity sensor, we measured the stress–strain curve and relaxation modulus (Figure 3c) using a differential mechanical analyzer (DMA). The relaxation modulus varied from 3300 to 2000 MPa when the fiber was stretched to 1.6%. Also, a 700 g weight was lifted by a single-fiber capacitive humidity sensor. These tests indicate that the fiber humidity sensor is strong enough for wearable application.

### 3.2. Measuring Sensor Capacitance and Effective Dielectric Constant

**Figure 4:**
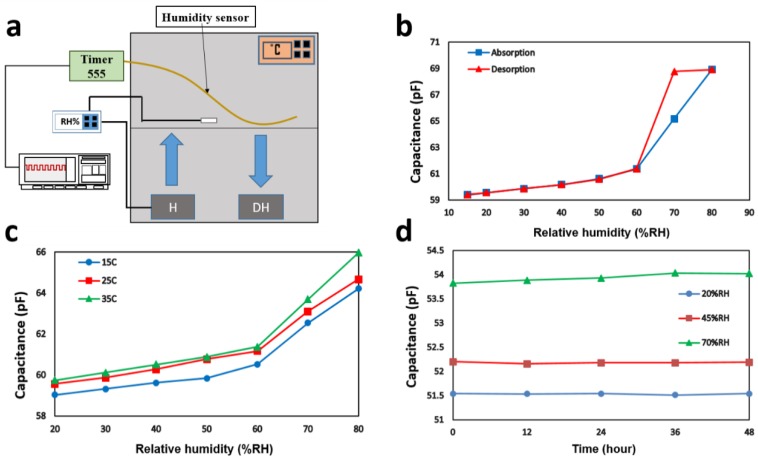
Fiber capacitive humidity sensor characterization. (**a**) Diagram of humidity sensor measurement set-up. (**b**) Capacitance variations versus relative humidity (RH) levels from 15% RH to 80% RH at room temperature. (**c**) The performance of the fiber humidity sensor at 15, 25, and 35 °C. (**d**) Long-term stability of the humidity sensor under RH levels of 20%, 45%, and 70% at 25 °C for 48 h. The capacitance change was extremely small during 48 h.

We used a custom-made testing chamber as shown in Figure 4a to characterize the response of the humidity sensor. In particular, we used an environmental chamber that was capable of controlling the temperature. A commercial digital air humidity controller sensor with the ability of measuring humidity to an accuracy of 3% over the range of 1% to 99% RH was used to monitor and control the humidity inside the chamber. A humidifier was controlled by the humidity controller sensor, and two dehumidifiers were placed in the chamber to maintain the humidity level. To allow us to measure the capacitance variation, we placed the fiber humidity sensor with a length of 150 cm inside the chamber, and connected the fiber humidity sensor to a CMOS 555 that was further connected to an oscilloscope. To measure the capacitance, we used an analog timer circuit which had an output of frequency (*f*). The schematic of the circuit is shown in Figure S2 (Supplementary Materials). The output of this circuit was a frequency that could be read by connecting the circuit to the oscilloscope or similar devices. The frequency is inversely proportional to the capacitance through the following relationship:

(1) $$f=\frac{1}{\left(C\left(R_{1}+2R_{2}\right)\ln\left(2\right)\right)}$$

where *R*_1_ and *R*_2_ are equal to 56 and 470 kΩ, respectively, C is the capacitance of the fiber sensor. We then calculated the capacitance by reading the period from the oscilloscope. To evaluate the performance of the sensor in terms of the sensitivity and hysteresis, the humidity level of the chamber was cyclically increased and decreased from 20% to 80% RH by an increment of 10%.

The capacitance per unit length of an ideal capacitator with two parallel wires is

(2) $$C=\frac{\pi \varepsilon_{eff}\varepsilon_{0}}{ln\left(\frac{d}{a}\right)},\;d\gg a$$

where *d* is the spacing between the center of the two wires, and *a* is the radius of the metal wire. $\varepsilon_{0}$ is the absolute dielectric permittivity of classical vacuum,$\;\varepsilon_{eff}$ is the effective dielectric constant of the polymer dielectric, which we discuss below. Here, *a* = 0.127 mm and *d* = 0.475 mm, measured from the optical image of the fiber tip cross-section.

We used the following formula to find the value of $\varepsilon_{eff}$ [61]:

(3) $$\frac{\varepsilon_{eff}-\varepsilon_{m}}{\varepsilon_{eff}+\beta \varepsilon_{m}}=f\frac{\varepsilon_{air}-\varepsilon_{m}}{\varepsilon_{air}+\beta \varepsilon_{m}}$$

where $\;\varepsilon_{air},\;\varepsilon_{m},\;$ and $\varepsilon_{eff}\;$ are the dielectric constants of air and the polymer matrix, and the effective dielectric constant of the porous material, respectively.$\;\varepsilon_{air}$ = 1, whereas $\;\varepsilon_{m}=3.15\;$ for PEI according to the material’s data sheet (RH = 55%, *T* = 23 °C). Additionally, f is the porosity of the porous material, which was estimated to be 48% by measuring the porous area percentage in the optical images of the fiber using Image J software. β is the structure factor with a value of 1 for the through-hole structure, and 3 for the closed-pore structure. For this study, we assumed β=1 (through-hole model) because of the unidirectional fiber drawing characteristics.

Using Equation (2), we found the $\varepsilon_{eff}\;$ of the sensor to be 1.89, which was significantly smaller than the dielectric constant of pristine PEI (3.15). This drastic reduction in the effective dielectric constant was expected due to the incorporation of air inside PEI. Plugging the $\varepsilon_{eff}\;$ value into Equation (1), we calculated the capacitance of a 1-m-long fiber humidity sensor to be 39.85 pF, which was close to our measured capacitance value of 36.06 pF/m at RH = 55% and *T* = 23 °C. Therefore, Equation (2), coupled with the assumption of the through-hole model, was a reasonable model to evaluate the effect of porosity on the effective dielectric constant and the capacitance variation in our porous PEI fiber.

It is worth mentioning that, to further control the generation of porosity in the fiber capacitive humidity sensors, we can utilize polymer solution and control its phase separation during thermal drawing, as shown in a recently published work by Grena et al. [35].

## 4. Discussion

### 4.1. Sensor’s Sensitivity

Figure 4b shows the hysteresis loop of the sensor at 25 °C. By increasing the RH level of the chamber, the output capacitance value of the sensor increased. This was because the dielectric constant of water ($\varepsilon_{water}=80$) is much higher than that of the PEI ($\varepsilon_{PEI}=3.15$) at room temperature. When the dielectric layer absorbs water from the environment, the capacitance of the polymer increases. The response, however, is nonlinear. The slope of the curve (the sensitivity) had two different values for low and high humidity levels. In particular, in the range of lower humidity levels (15%–60%), the sensitivity was lower than that in higher humidity levels (60%–80%).

The adsorption process of water molecules on a solid surface takes place through chemisorption at lower RH levels and physisorption at higher RH levels. In the chemisorption process, a monolayer of water molecules is formed on the walls of the pores and attached to solid molecules through chemical forces. When the RH level increases, physical adsorption takes place, in which water molecules aggregate layer by layer through van der Waals forces, and attach themselves to the solid molecules to form a multi-layer of water molecules on the solid surface. Due to the weak interactions between water molecules, the multilayered water molecule formed via physisorption diminishes very fast as RH level decreases [62]. However, by incorporating porosities in the solid, capillary condensation may take place at lower RH levels in the pores with a radius up to $r_{K}$, which is described by the Kelvin Equation.

(4) $$r_{K}=\frac{2M\gamma \;cos\theta }{R\rho T\;ln\left(\frac{P_{s}}{P_{0}}\right)}$$

where $\frac{P_{s}}{P_{0}}$ is the relative pressure, *M* and ρ are the molecular weight and density of water, respectively, γ is the surface tension, *R* is the gas constant, *T* is the absolute temperature, and θ is the contact angle.

Vapor condensation normally occurs at high RH levels; however, in our case, capillary condensation took place at low RH levels (60%–70%) due to the porous structure of the polymer, resulting in an abrupt increase in the output capacitance at 70% RH. The significant difference between the permittivity values of water droplets ($\varepsilon_{water}\approx 80$) in comparison with that of water vapors ($\;\varepsilon_{air}\;$= 1) caused the abrupt increase of the capacitance of the sensor at 70%, where the capillary condensation effect occurred [38]. Therefore, the sensitivity at lower humidity levels (15% to 60%) was lower than that at higher humidity levels (60%–80%).

In order to have a more precise estimation of the sensitivity and hysteresis of the sensor, we calculated the sensitivity in the two regimes. The sensitivity *S* of the humidity sensor is defined as

(5) $$s=\frac{\Delta C}{\Delta RH}\;\;\left(pF/\%\;RH\right)$$

where ΔC is the capacitance change of the fiber humidity sensor. According to Equation (4), the capacitance sensitivity was 0.04 *p*F/% RH in the first linear regime (15%–60%), and 0.38 pF/% RH in the second linear regime (60%–80%). This result indicates a significantly higher sensitivity in the second linear regime compared with the first one.

Furthermore, the hysteresis of the humidity sensor can be characterized by *H*, defined as

(6) $$H=\frac{C_{D}-C_{A}}{S}\;\;\left(\%\;RH\right)$$

where *C*_D_ and *C*_A_ denote the capacitance value in desorption and absorption processes, respectively. Our RH sensor exhibited a maximum hysteresis of 9.45% RH at 70% RH. In the lower RH level (15%–60% RH), the hysteresis was very low, with a maximum hysteresis of 0.8% RH at 50% RH.

### 4.2. Temperature Dependence and Stability

Figure 4c shows the relationship between the capacitance of the sensor and relative humidity in three different temperatures: 15, 25, and 35 °C. For each temperature, capacitance variation of the sensor in the entire humidity range followed almost the same trend, showing the strong temperature stability of this sensor. In addition, we observed that, by increasing the temperature, the value of the capacitance slightly increased. Considering that the thermal movement of molecules plays a critical role in the polarization of the material, by raising the temperature, stronger thermal movements of molecules can result in higher polarization and, consequently, higher magnitude of capacitance [27]. Because the slopes of all curves in both linear regimes were almost the same, we conclude that our capacitance humidity sensor’s sensitivity is independent of the temperature variation.

In order to investigate the long-term stability of the sensor, we placed the sensor with a length of 120 cm in the chamber under ambient relative humidity (20%, 45%, and 70%) at 25 °C for 48 h. Figure 4d shows that the capacitance values of the sensor remained very stable for both low RH (20% and 45%) and high RH (70%).

## 5. Conclusions

In this study, we used a high-throughput convergence fiber drawing method to fabricate a novel fiber capacitive humidity sensor with porous polymer as its sensing layer and metal wires as its electrodes. The porosity is generated by evaporation of the absorbed water in the polymer preform during thermal drawing. The pore size and uniformity are controlled by the water weight percentage of the preform and the cooling rate during fiber drawing. Our fiber sensor exhibits good flexibility with reliable sensing performance after 20 cycles and at a bending radius as small as 4 mm. This new fiber capacitive humidity sensor demonstrates a good sensitivity of 0.38 pF/% RH in the range of 70%–80% RH, as well as a maximum hysteresis of 9.45% RH at 70% RH. It has a very low hysteresis at lower RH levels (15%–60% RH) with a maximum hysteresis of 0.8% RH at 50% RH. Furthermore, the sensor showed a good temperature-independent performance and long-term stability. In the network of pores formed during the thermal drawing process in the polymer dielectric of the sensor, the pores satisfying the Kelvin relationship induced capillary condensation, which resulted in capillary condensation at lower RH levels (in our case, at 70%RH). Consequently, the sensitivity of the sensor was significantly enhanced in the relative humidity region of 60%–80% RH.

This work demonstrates the first fiber-based humidity sensor fabricated using convergence fiber drawing. This convergence fiber drawing method allows for scalable production of multimaterial fiber devices that integrate highly flexible and versatile polymer materials with highly conductive metals, which can open many new opportunities for fabricating flexible sensor devices.

## Supplementary Materials

The following are available online at https://www.mdpi.com/2073-4360/11/12/1985/s1.
