# Supplementary material for: Scalable Fabrication of Highly Flexible Porous Polymer-Based Capacitive Humidity Sensor Using Convergence Fiber Drawing
Source: Polymers (Basel). 2019 Dec 2;11(12):1985. doi: 10.3390/polym11121985 (PMC6960705; doi:10.3390/polym11121985)
Supplement: Supplementary file 1 [file polymers-11-01985-s001.pdf]

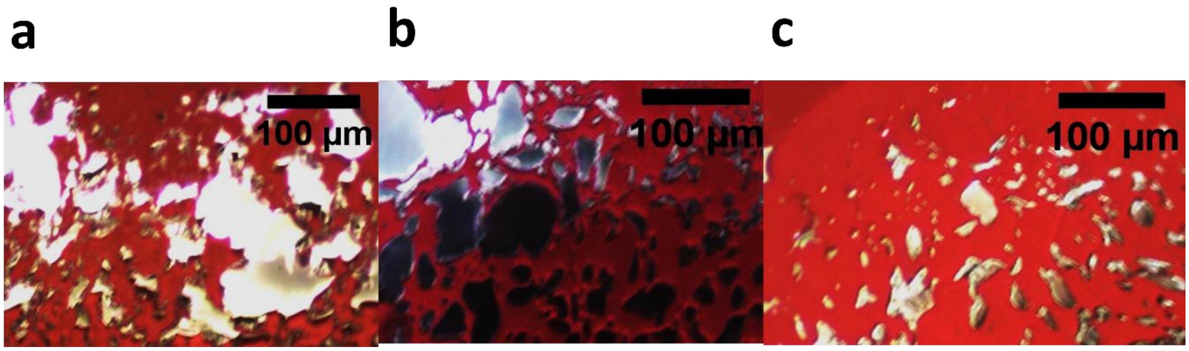

Figure S1. Optical image of the cross sections of the porous fibers drawn by TDP at the same middle zone temperature of 342 ˚C and different bottom zone temperatures of **a**) 195 ˚C, **b**) 180 ˚C, **c**) 150 ˚C

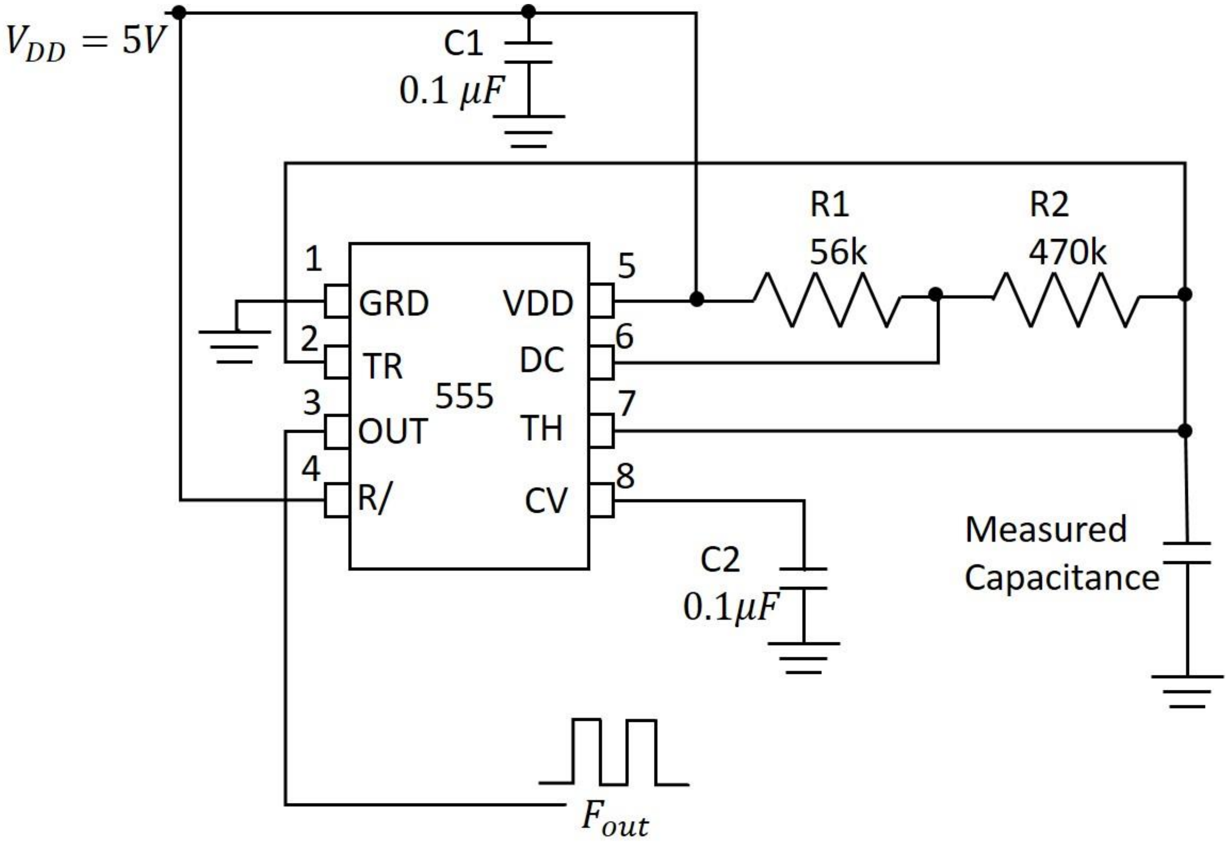

Figure S2. The schematic of the circuit.
